# The STROGHAT study protocol: An intervention study to evaluate safety, effectiveness and feasibility of treating gambiense HAT seropositive subjects with acoziborole.

**DOI:** 10.12688/openreseurope.19077.1

**Published:** 2025-01-24

**Authors:** Elena Nicco, Veerle Lejon, Erick Mwamba Miaka, Dieudonné Mumba, Alain Mpanya, Charles Kambo, Digas Ngolo, Wilfried Mutombo, Stéphane Hugonnet, Sandra Rembry, Craig Tipple, Raquel Inocencio Da Luz, Rian Snijders, Catiane Vander Kelen, Stijn Rogé, Nick Van Reet, Antoine Tarral, Paul Verlé, Epco Hasker

**Affiliations:** 1 Institute of Tropical Medicine Antwerp, Antwerp, 2000, Belgium; 2 French National Research Institute for Sustainable Development IRD, CIRAD, University of Montpellier, Montpellier, 34398, France; 3 Programme National de Lutte contre la Trypanosomiase Humaine Africaine, Kinshasa, Democratic Republic of the Congo; 4 Av. De la Démocratie, Institut National de Recherche Biomédicale, Kinshasa, Democratic Republic of the Congo; 5 Drugs for Neglected Diseases initiative Kinshasa, Kinshasa, Democratic Republic of the Congo; 6 Drugs for Neglected Diseases initiative, Geneva, Geneva, Switzerland; 7 Independent Consultant, Geneva, 1202, Switzerland; 8 Independent Consultant, Antwerp, 2000, Belgium

**Keywords:** Human African Trypanosomiasis, Elimination, Democratic Republic of Congo, Acoziborole.

## Abstract

**Background:**

Coordinated efforts in the control of gambiense human African trypanosomiasis (gHAT) have significantly reduced its endemicity. WHO targets interruption of transmission by 2030. However, challenges remain, including low sensitivity of the current parasitological confirmation tests, leaving a potential human reservoir untreated. Acoziborole, a single-dose oral treatment, effective in both disease stages with a good safety profile, offers the potential of treatment of parasitologically negative gHAT seropositive subjects, which could improve diagnostic sensitivity. The STROGHAT study aims to evaluate whether this approach can lead to elimination of *T.b. gambiense* from its human reservoir, and to provide further safety data on acoziborole. It also includes a costing analysis and a prospective evaluation of the performance of the screening and diagnostic tests used.

**Methods:**

STROGHAT is a one-arm epidemiological study, with a nested phase IIIb, one-arm, open label, non-randomized, multicentre clinical trial. It will be implemented over four years in the endemic region of the Equateur North, in the Democratic Republic of Congo. For the first three years, parasitologically negative gHAT seropositive subjects will be treated with acoziborole, while parasitologically confirmed cases will receive standard of care. Individual follow-up needs and accurate prevalence estimate will be based on immunological and molecular tests performed for all gHAT screening test seropositive subjects at a reference laboratory. In the fourth year, standard procedures will resume, and a prevalence survey will assess whether interruption of transmission has been achieved.

**Discussion:**

The manuscript outlines the study background, objectives and methods while discussing its strengths and challenges. If successful, the STROGHAT study will provide critical evidence on the effectiveness, safety and feasibility of the new strategy, and inform future elimination strategies.

**Clinical trial registration:**

NCT06356974. Date of registration: April 4, 2024.

## Plain Language Summary

The number of people getting sleeping sickness in central and western Africa has been going down in recent years. Since the trypanosome species causing the disease in these countries has a human reservoir, the World Health Organization hopes, by 2030, to eliminate its transmission. Simple screening tests exist but diagnostic confirmation is challenging. This hampers treatment coverage but was always considered necessary in view of complexity and toxicity of the older treatment regimens. Now a new oral medication becomes available. It has minimal side effects, and only requires one dose to cure sleeping sickness. This could allow introducing presumptive treatment, based on a positive screening test only.

In the STROGHAT study, researchers will evaluate if giving this new drug to everyone who tests positive to a serological screening test for sleeping sickness, without need for further confirmation, could help getting rid of the disease. The study will take place in a region in the north of the Democratic Republic of the Congo. Researchers will also check for any side effects of the new drug. Additionally, they will evaluate new diagnostic tests that would enable confirmation of the disease in a remote laboratory at a later timepoint, and look at the cost of this approach.

If the study is successful, this innovative approach could be applied to eliminate sleeping sickness transmission in other affected regions and countries.

## Introduction

Gambiense Human African trypanosomiasis (gHAT) is a parasitic disease endemic in Central and West Africa, caused by *Trypanosoma brucei gambiense* and transmitted from person to person by the tsetse fly vector ^1^. Animals can be infected, but to date there is no convincing evidence about their role in transmission ^2^. The disease has a chronic course with first stage caracterized by aspecific symptoms, followed by a second stage with neurologic symptoms. Without treatment, death will almost always occur within a few years. More than half of global gHAT cases are diagnosed in the Democratic Republic of Congo (DRC) ^3^. Control measures are based on serological mass screening of at-risk populations (active screening); serological screening of symptomatic people seeking care at health facilities (passive screening); parasitological examination of seropositives and treatment of parasitologically confirmed gHAT cases and vector-control ^4^. In 1991, Simarro *et al*. demonstrated that active serological screening of at-risk-populations, followed by parasitological confirmation and treatment of confirmed cases, was the most cost-effective approach for gHAT control ^5^. In the DRC, populations living in gHAT endemic villages are screened yearly by mobile teams until no cases are reported for three consecutive years. These villages are then screened once more, in the two years after the last case was reported. If no further cases are found, transmission is assumed to be interrupted ^6^. WHO defines absence of transmission as zero parasitologically confirmed human cases of gHAT for five consecutive years ^6^.

In recent years, thanks to sustained control measures, the prevalence and incidence of the disease are at an all-time low. The World Health Organization (WHO) aims for interruption of transmission by 2030 ^7^. However, the current approach, restricting treatment to parasitologically confirmed gHAT cases only, seems insufficient for achieving interruption of transmission. Up to 50% of prevalent cases may remain undetected or untreated due to significant losses throughout the screening, confirmation and treatment cascade ^8^. To overcome this challenge, Simarro *et al*. have already demonstrated in an island setting that interruption of transmission can be achieved by treating not only parasitologically confirmed cases, but also all individuals who tested positive solely in serological screening tests ^9^. At that time, treatment options were still limited to complex and toxic treatments, with a mandatory lumbar puncture for disease staging, impeding large-scale implementation of programmes promoting extended gHAT treatment.

Recently, acoziborole ( Table 1), a single dose oral treatment, has successfully completed phase 3 evaluation, showing a treatment success rate for both disease stages comparable to nifurtimox eflornithine combination therapy (NECT), the current standard of care for stage 2 ^10^. As acoziborole can cure both stage 1 and stage 2, the lumbar puncture required for staging would no longer be necessary. Data from treating patients with confirmed gHAT have demonstrated a favorable safety profile and reviews by Independent Data Monitoring Committees (IDMC) of a recently completed safety study in non-parasitologically confirmed gHAT screening test seropositive subjects (Clinicaltrials.gov NCT05256017), have not identified additional risks ^10^.

**Table 1.** Summary of available evidence for acoziborole.

Trial name	Trial phase	Trial characteristics	Number of volunteers/patients enrolled	Findings
**DNDi-OXA-001** EudraCT n°2011- 004639-30 Tarral A, *et al.* (2023) doi: 10.1007/s40262-023-01216-8.	I	Randomized, double blind, placebo controlled (part 1). Three parts: I) single ascending doses (20–1200mg); IV) impact of activated charcoal; VI) relative bioavailability of a new tablet formulation	128 healthy males of sub-saharan African origin (18–45 yo): 102 acoziborole (84 in part 1, 6 in part IV and 12 in part IV); 26 placebo	Safety – no signal observed Tolerability – no severe adverse events Pharmacokinetics & Pharmacodynamics: active exposure at 960 mg dose
**DNDi-OXA-03-HAT** NCT04270981	I	Single center, open-label, single-group, non-randomized, single oral study to assess mass balance recovery, pharmacokinetics, metabolite profile and metabolite identification of acoziborole	6 healthy Caucasian male participants	mass balance recovery – fecal excretion is predominant route Pharmacokinetics – similar to OXA001 Metabolite profile and metabolite identification - oxidative deboronation and mono/di- oxidation, and glucuronidation, desaturation for the successive metabolites.
**DNDi-OXA-07-HAT** NCT05947604	I	Single center, open-label, non- randomised, three-treatment, two period Single dose acoziborole, sequential co-administration midazolam and dextromethorphan	39 healthy male participants	Pharmacokinetics & drug interaction: acoziborole is a strong CYP2D6 inhibitor and a strong CYP3A4 inducer
**DNDi-OXA-02-HAT** NCT03087955 Betu Kumesu, Victor Kande *et al.* (2023)	II/III	Multicenter, open-label, prospective phase II/III pivotal study to assess the efficacy and safety of acoziborole Single dose 960 mg under fasting conditions	208 participants with HAT due to *T. b. gambiense* (male and female ≥ 15 yo, sub-Saharan Africa)	Efficacy: treatment success rate at 18 months in patients with late-stage HAT: 95.2% (n/N; CI 95%, 91.2 - 97.7) for the mITT set, and 98.1% (159/162; CI 95%, 95.1 - 99.5) for the evaluable patients. In early- and intermediate-stage HAT patients, the success rate at Month 18 in the mITT set was 100% (n/N; 95% CI, 94.1 – 100). Safety – 27 SAE not related to acoziborole
**DNDI-OXA-04-HAT** NCT05256017	II/III ONGOING	Multicenter, randomized, double blind, placebo- controlled (3:1 Active:Placebo), Single dose 4 months follow-up Tryp-skin sub-study (725 participants)	1208 participants gHAT screening test seropositive, not parasitologically confirmed	Safety & Tolerability Pharmacokinetics (trypskin sub- study) Study data under analysis
**DNDI-OXA-05- HAT** NCT05433350	II/III ONGOING	Open-label, non-randomized, single arm, multicenter Single dose Fasting conditions	Children (1–14 yo) with HAT due to *T. b. gambiense*	pharmacokinetics, efficacy, safety, tolerability Study recruitment ongoing

The table gives a list of completed and ongoing trials, together with their phase, the main characteristics of the trial and of enrolled participants and summarizes the main results. The STROGHAT study is not included in the table. Yo: years old; CI: Confidence Interval; mITT: modified intention to treat population; SAE: Serious Adverse Event.

The characteristics of acoziborole (single dose, oral, favorable safety profile and highly effective) will allow treatment of all non-parasitologically confirmed gHAT screening test seropositive subjects, provided the favorable safety profile of acoziborole is confirmed when tested on a larger population. This will avoid losses due to the complexity and imperfect sensitivity of the current diagnostic algorithm, and we hypothesize that this would lead to the elimination of the *T.b. gambiense* parasite from its human reservoir. If confirmed, this new molecule not only has the potential to fundamentally change the perspective for gHAT management at the individual level, but could also revolutionize gHAT control and elimination strategies at the population level. To test this hypothesis, the Stop Transmission of gHAT (STROGHAT) study aims to demonstrate that systematically treating all those seropositive for gHAT can lead to interruption of transmission, even on a mainland setting, and provide further evidence of the safety of acoziborole ^9^.

## Study protocol

### STROGHAT study objectives

The STROGHAT study is an epidemiological study, with a nested clinical trial. The one-arm epidemiological study aims at demonstrating zero prevalence of *T.b. gambiense* infection after three years of implementation of a so-called 'screen and treat' strategy with acoziborole. It also includes a costing analysis of the screen and treat strategy, and a prospective evaluation of the performance (specificity and positive predictive value) of the screening and diagnostic reference laboratory tests used.

The nested STROGHAT clinical trial is a one-arm, open label, non-randomized, multicentre, phase IIIb study to assess the safety of acoziborole in participants with non-parasitologically confirmed gHAT screening test seropositivity. The STROGHAT study will complement existing safety data (DNDI-OXA-04-HAT, clinicaltrials.gov NCT05256017), increasing the chances of detecting any potential uncommon or/and rare treatment related severe adverse events. The SPIRIT checklist is available for further details ^11, 12^.

### The study setting & study population

**Figure 1.**
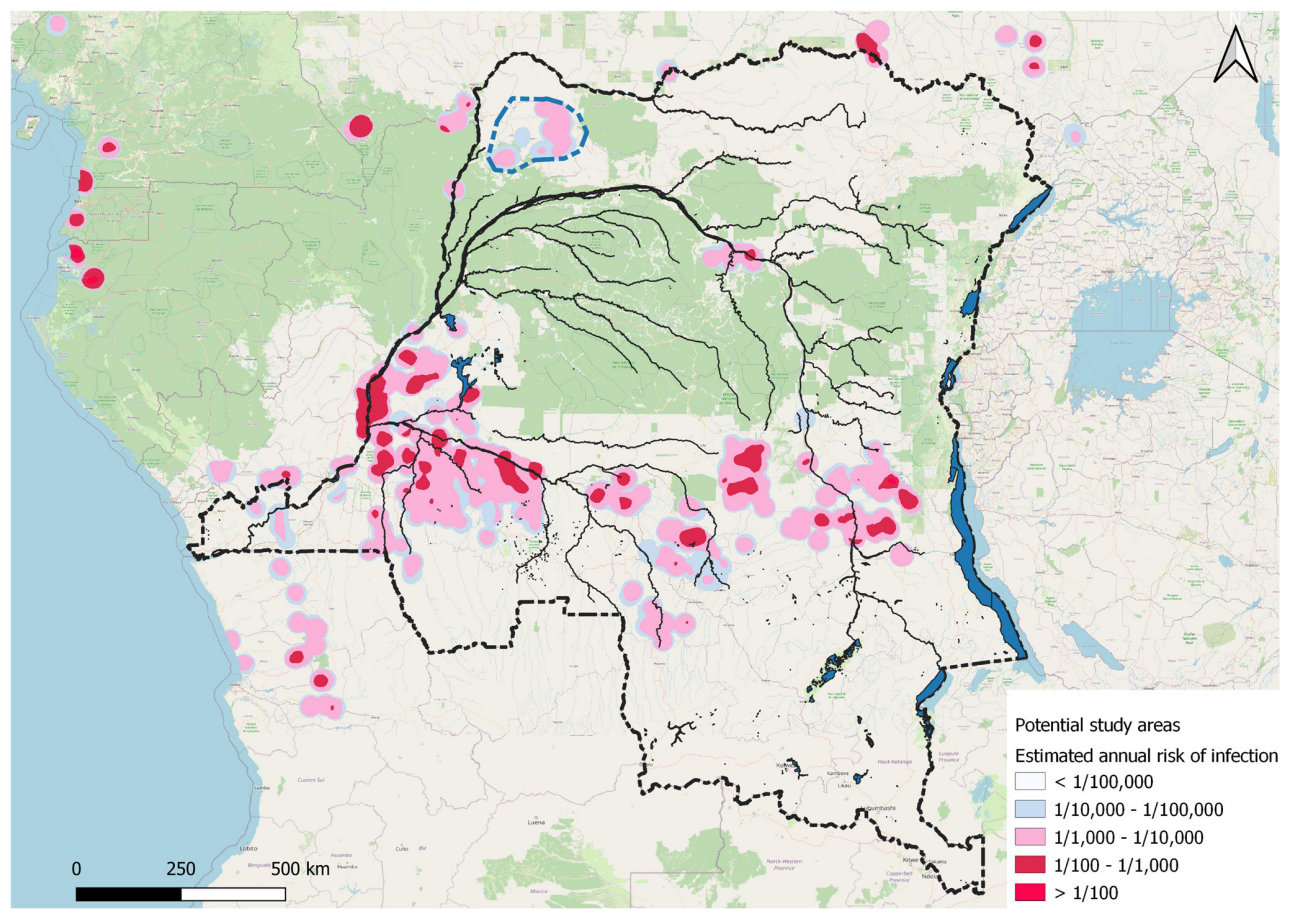
Moderate to high gHAT risk clusters in DRC (2017–2021). Up to 20 potential intervention areas in DRC with an annual risk of gHAT infection above 1 per 10,000 population are shown. Moderate to high gHAT risk clusters are shown in different colours. The risk of infection is based upon cases reported between 2017 and 2021 in DRC.

The study will be implemented in the DRC. An important feature of gHAT is its focal distribution. For the STROGHAT study we identified, based on the previous five years’ case notifications (2017–2021), 20 potential intervention areas in the DRC with an annual risk of infection above 1 per 10,000 ( Figure 1) which can, according to WHO criteria, be considered as moderate to high risk areas (respectively, more than one HAT case per ten thousand or per thousand persons at risk per year) ^13– 16^.

**Figure 2.**
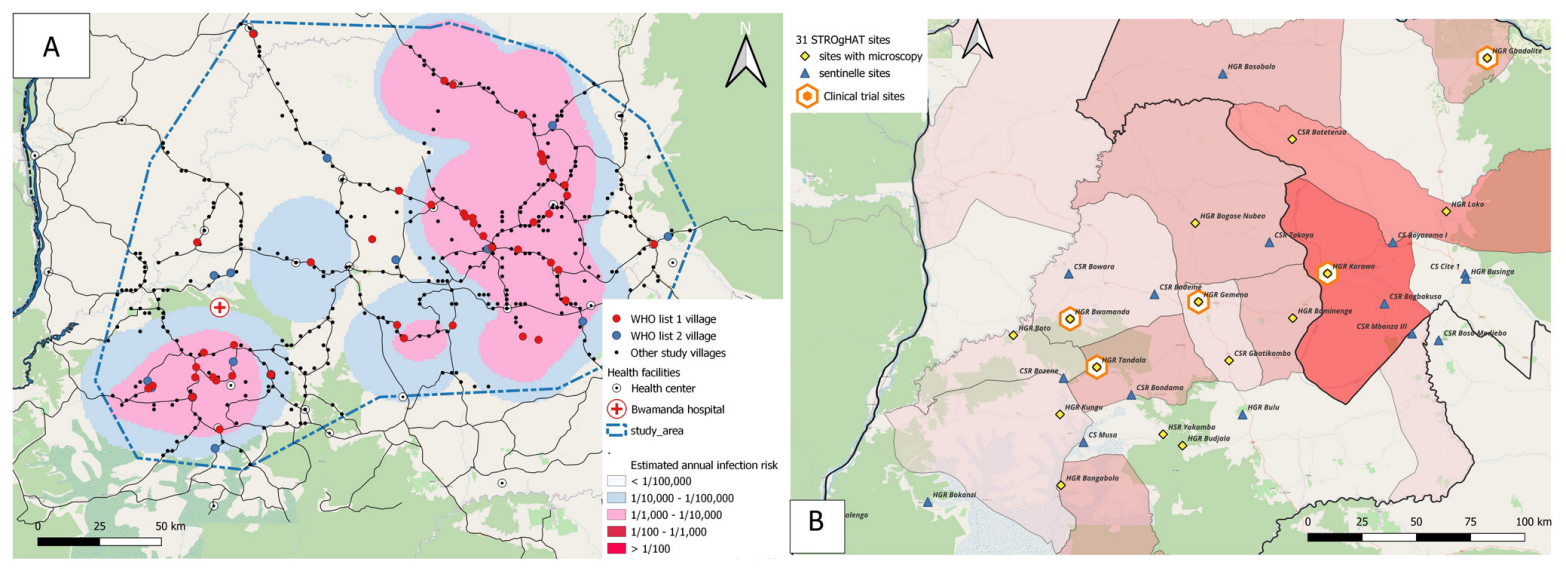
Equateur North province, DRC; **A**) STROGHAT study area; **B**) STROGHAT study sites. In Figure **2A** the gHAT risk cluster selected for the study is outlined by the blue dotted line and is made up of two moderate risk clusters (1/1,000-1/10,000 population). The study area includes 94 villages requiring active screening (WHO list 1 and list 2 villages) and 309 villages with no recently reported cases (last 5 years). Figure **2B** shows the 31 STROGHAT clinical trial sites.

Among those, we searched for an area that is accessible and that is surrounded by areas of low to negligible risk (less than 1 HAT case annually per 10,000 persons at risk). Security concerns were also taken into account. Based on these criteria the Equateur Nord region in the North-West of the DRC was selected. The intended study area ( Figure 2A) is made up of two moderate risk clusters (annual infection risk between 1 in 1,000 and 1 in 10,000) in which there are 94 villages that would require active screening plus 309 villages from which no gHAT cases have been reported over the past five years. The WHO's elimination strategy ^4^ recommends conducting annual village screenings in areas with ongoing transmission until no new cases have been detected for three consecutive years (‘list 1’ villages). Once this is achieved, one additional round of screening is recommended over the following two years (‘list 2’ villages). The 94 villages have an estimated population of approximately 175,000. This is the population to be screened in the first year of the study. If no new gHAT cases are found, villages from list 2 will not be screened the following years. Villages on list 1 that have no cases for three consecutive years will be moved to list 2. If new cases are detected in villages not previously reporting gHAT or in former list 1 or list 2 villages, these villages will be included in the WHO list 1 and will be screened. Such cases could be detected through passive screening which is provided at existing sentinel sites for gHAT. The total population of the study area is estimated to be between 2 and 2.5 million. During the first study year, all villages in the study area will be mapped and more accurate population estimates will be obtained.

All age and sex groups will be included in the epidemiological study, while specific inclusion and exclusion criteria will be applied for the clinical trial.

### STROGHAT study design

The total duration of the STROGHAT study will be four years. Alongside passive case finding at 31 sentinel sites in the study region ( Figure 2B), each year, four mobile teams will perform active case finding in villages that reported cases within the past three years (list 1) or three to five years ago (list 2).

For all gHAT screening test seropositive subjects, a venous blood sample will be collected on the spot. Heparinized venous blood (0.5 ml) will be used for parasitological confirmation using the mini anion exchange centrifugation technique (mAECT, INRB, Kinshasa) ^17^. Dried blood spots (DBS) (12 spots of 50 µl on 2 Whatman qualitative filter papers, Grade 4 circles, diam. 90 mm), and 1 ml of blood stabilized on 1 ml RNA/DNA shield solution 2x (Zymo Research, R1200-125) will be sent to the national reference laboratory for sleeping sickness, at the Institut National de Recherche Biomédicale (INRB), for post-hoc confirmation by highly specific immunological and molecular tests. For study purposes, we will maintain on-site parasitological confirmation. Although phase 3 data support its use in this trial from a safety perspective, acoziborole has not yet been formally approved for the treatment of gHAT, therefore, anyone confirmed by on-site parasitology will be treated with standard of care, at present fexinidazole, NECT or pentamidine ^18^.

All screened individuals seropositive for gHAT but testing negative for trypanosomes in the parasitological examinations on the spot will be enrolled in the clinical trial and receive acoziborole on-site, provided they meet the clinical trial selection criteria, and provide informed consent. Dried blood spots and blood samples on RNA/DNA shield solution collected in the field will be sent to INRB for immunological testing with ELISA/ *T.b.gambiense* ^19^ and immune-trypanolysis ^20^ and molecular testing with *Trypanozoon*-RT-qPCR multiplex ^21, 22^ respectively. In case study participants test positive in the post-hoc tests and are enrolled in the clinical trial, a sample of venous blood will be collected at month 4 post-administration of acoziborole to evaluate parasite clearance and assess the need for additional treatment.

The enrolment period for the clinical trial will last three years. During the fourth year of the intervention (assumed to be 2027), no further enrolment for the clinical trial will occur, but active and passive case finding will be continued according to the WHO and national guidelines in the DRC. In the fourth study year a random sample of villages reporting gHAT cases prior to five years ago (historic villages) will also be screened, in addition to the endemic villages on WHO list 1 and 2. In the year-four survey, seropositive individuals will be managed according to the national protocol and will not anymore receive any treatment unless they are parasitologically confirmed.

### Outcome measures

The primary endpoint of the STROGHAT epidemiological study is the prevalence of confirmed gHAT cases, that is the number of confirmed gHAT cases divided by the number of screened individuals in the fourth study year (2027). A confirmed gHAT case is defined in the STROGHAT study either at the point of care by a positive parasitological test or at the reference laboratory by a combination of a positive immunological test and a positive molecular test ( Table 2).

**Table 2.** STROGHAT study tests interpretation.

	Test	Results	Case yes/no (prevalence measurement)	Treatment	4 months sample	Test accuracy calculation = specificity
**Screening**	**CATT**	Pos	Inconclusive Yes, if at least one parasitology or both confirmatory test are also positive Otherwise No	Yes (if parasitology pos = SOC; if parasitology neg = inclusion in study part B)	Yes (if ≥1 confirmatory test pos & inclusion in study part B)	Numerator: all CATT negatives Denominator: all CATT positives with negative parasitological test + all CATT negatives
		Neg	No	No	No	
	**HAT Sero *K*-Set**	Pos	Inconclusive Yes, if at least one parasitology or both confirmatory test are also positive Otherwise No	Yes (if parasitology pos = SOC; if parasitology neg = inclusion in study part B)	Yes (if ≥1 confirmatory test pos & inclusion in study part B)	Numerator: all RDT negatives Denominator: all RDT positives with negative parasitological test + all RDT negatives
		Neg	No	No	No	
**Parasitology**	**Combined mAECT, ** **lymph and CSF ** **results (CSF only if ** **LP indicated)**	Pos	Yes	Yes (SOC)	No	Reference test
		Neg	No (only if immunological AND molecular confirmatory test are both positive)	Yes (if inclusion in study part B)	Yes (if INRB confirmatory test pos & inclusion in study part B)	Reference test
**Confirmatory**	**Molecular:** ***Trypanozoon*-RT-** **qPCR multiplex**	Pos	Yes if serological AND molecular confirmatory test pos	Unrelated to treatment	Yes (if inclusion in study part B)	Numerator: all qPCR negatives Denominator: all qPCR negatives + all qPCR positives with negative parasitological test
		Neg	No (only if parasitology is pos)	Unrelated to treatment	Only if another INRB confirmatory test pos & inclusion in study part B	
	**Immunology: ** **Immune-** **trypanolysis**	Pos	Yes if immunological AND molecular confirmatory test pos	Unrelated to treatment	Yes (if inclusion in study part B)	Numerator: all TL negatives Denominator: all TL negatives + all TL positives with negative parasitological test
		Neg	No (only if parasitology is pos or molecular and another immunological confirmatory test pos)	Unrelated to treatment	Only if another INRB confirmatory test pos & inclusion in study part B	
	**Immunology: ** **ELISA/ *T.b. *** ***gambiense***	Pos	Yes (if immunological AND molecular confirmatory test pos)	Unrelated to treatment	Yes (if inclusion in study part B)	Numerator: all ELISA negatives Denominator: all ELISA negatives + all ELISA positives with negative parasitological test
		Neg	No (only if parasitology is pos or molecular and another immunological confirmatory test pos)	Unrelated to treatment	Only if another INRB confirmatory test pos & inclusion in study part B	

The Table summarizes how the results of the different screening and diagnostic tests performed in the study are used to calculate the prevalence of HAT (case definition), to determine treatment eligibility in the study, to determine the need for biological follow-up and for evaluating the diagnostic specificity. CATT: Card agglutination test for trypanosomiasis/ *T.b.gambiense*; SOC: standard of care; RDT: rapid diagnostic tests; mAECT: mini anion exchange centrifugation technique; CSF: cerebrospinal fluid; LP: lumbar puncture.

The gHAT prevalence with 95% confidence interval (CI) will be determined in the study year 1, 2, 3 and 4 among all participants screened in each respective year. We will additionally estimate the participation rate in the screening activities per age and sex group during the study period. In year 4, we will also measure the prevalence and 95% CI among a cluster random sample of approximatively 150,000 individuals from historic villages, and we will estimate a maximum prevalence in the joint population of approximately 300,000, assuming that we have oversampled those most at risk ^23^. We assume that for a prevalence of below 1 per 50,000 after three years of intervention, the reproduction number R _t_ will be below 1 and the disease will head towards extinction. However, the limitations of this design will not allow us to firmly conclude whether the expected reduction in prevalence can be attributed to the study intervention.

The primary endpoint of the nested clinical trial is to measure the proportion of participants who present treatment related emergent severe adverse events (severe related TEAEs). The clinical trial will additionally measure several exploratory endpoints, including the proportion of participants with: mild or moderate related TEAEs, any (mild, moderate, severe) TEAEs and serious treatment emergent adverse events (TESAEs). All clinical trial participants who received one tablet of acoziborole are defined as the study population to be included in the safety analysis. Changes in clinical signs and symptoms from baseline to the end of the study will also be described.

The epidemiological study will additionally collect cost data to perform a comprehensive analysis of the cost of the screen and treat strategy in the study area. The analysis will adopt a health-care provider’s perspective, focusing on costs incurred by the Ministry of Health and donors. Only costs directly related to the elimination strategy will be included, while research-related costs will be excluded from analysis. Financial and economic costs will be estimated by using a mixed costing approach. Analysis will account for both recurrent and capital costs. To assess robustness of findings, a sensitivity analysis will be conducted, applying discount rates of 0%, 5% and 10%. The analysis will also evaluate the influence of key cost drivers on the overall cost estimates.

We will also prospectively evaluate the specificity and positive predictive value of the screening and diagnostic tests used: Card agglutination test for trypanosomiasis/ *T.b.gambiense* (CATT, Institute of Tropical Medicine, Belgium) ^24^ and rapid diagnostic tests (RDTs, HAT Sero *K*-Set, Coris BioConcept, Belgium, K-15S2) ^25^ in the field, and ELISA/ *T.b.gambiense* ^19^, immune-trypanolysis ^20^ and *Trypanozoon*-RT-qPCR multiplex ^21, 22^ at the INRB reference laboratory level. If available, by end of the study, we will use the collected study specimens to retrospectively evaluate the performance of an inhibition ELISA currently being developed ^26^ and a new *T.b.gambiense* specific qPCR. The reference standard for specificity evaluation will be parasitological confirmation. Sensitivity will not be calculated because, in the context of low prevalence, only few positives are expected which will not allow for statistical inference.

### Epidemiological study implementation

**Figure 3.**
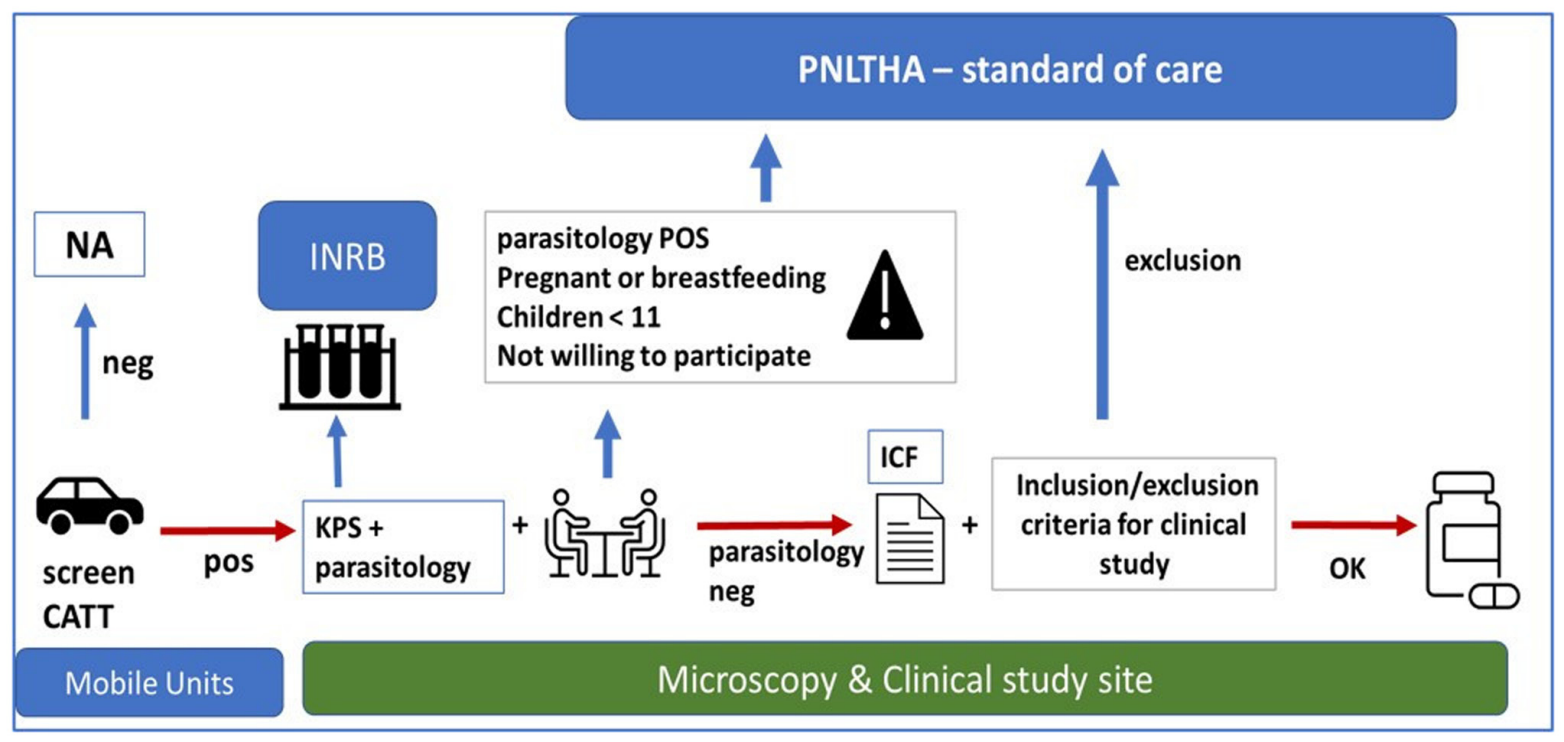
Flow chart of the study for active screening activities. The different steps of active screening in the STROGHAT study. CATT: Card agglutination test for trypanosomiasis/ *T.b.gambiense*; NA: not applicable; KPS: kit de prélèvement de sang (sampling kit); INRB: Institut National de Recherche Biomédicale (National Institut for Biomedical Research); PNLTHA: Programme National de Lutte contre la Trypanosomiase Humaine Africaine (National sleeping sickness control program); ICF: informed consent form.

Active screening of the endemic villages will be performed by dedicated screening teams from the national sleeping sickness control programme (PNLTHA), already active in the study area. Passive screening will involve 31 gHAT sentinel sites already operational in the region for a number of years ( Figure 2B). We will use the CATT on whole blood (Institute of Tropical Medicine, Belgium) in active screening and HAT Sero *K*-Set (Coris BioConcept, Belgium, K-15S2) in passive screening. No major changes from routine screening practice are planned. However, the three mobile teams will move together screening neighboring villages, with a single microscopy post set up in a nearby health facility, which will move as the screening teams move. On average, each mobile team is expected to identify less than five people who are seropositive per working day, who will be transferred from the village of screening to the microscopy-post for blood specimen collection, parasitological confirmation and gHAT treatment. Timely transfer of people who are seropositive will be ensured by car, with someone they trust to accompany them if they so wish. Minimal transfer delays will be ensured by setting up microscopy posts within a maximum travel time of 30 minutes, which is considered acceptable by the local health authorities involved in the study planning. Confirmation and treatment at the health facility could also guarantee better privacy compared to current practice, where confirmation takes place on site in the village during screening activities ( Figure 3).

**Figure 4.**
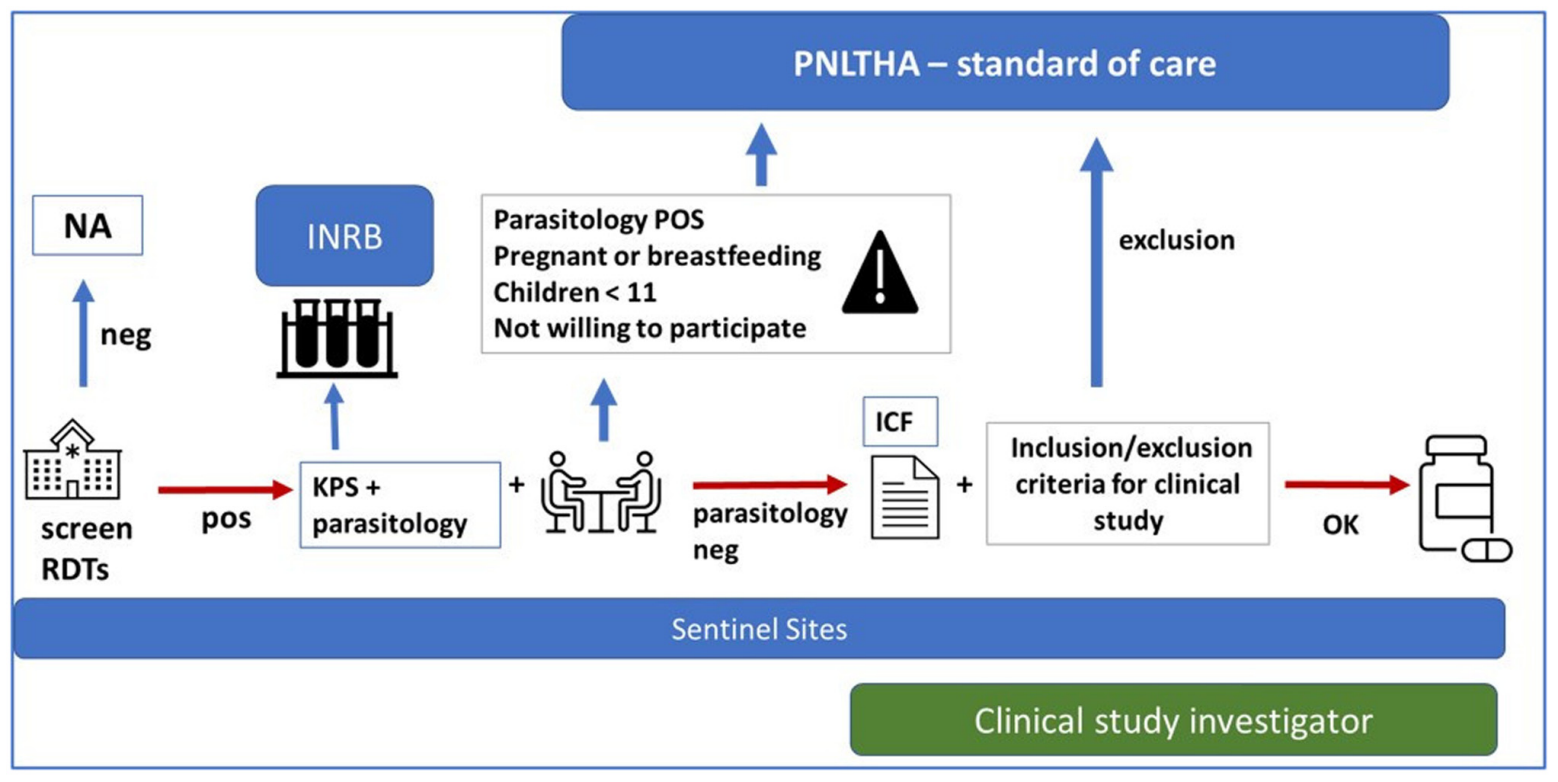
Flow chart of the study for passive screening activities. The different steps of passive case finding in the STROGHAT study are shown. Legend: RDTs: rapid diagnostic tests; NA: not applicable; KPS: kit de prélèvement de sang (sampling kit); INRB: Institut National de Recherche Biomédicale (National Institut for Biomedical Research); PNLTHA: Programme National de Lutte contre la Trypanosomiase Humaine Africaine (National sleeping sickness control program); ICF: informed consent form.

Passive case finding will be implemented in 31 fixed health facilities using a gHAT RDT. People who test RDT seropositive will be offered sample collection and parasitological confirmation either on site (15 sentinel sites with microscopy capacity), or upon referral to a nearby facility with microscopy capacity that is already part of the study network (16 sentinel sites without microscopy capacity). Among the 15 well equipped sentinel sites with full diagnostic capacity, five clinical trial sites have been selected. The five clinical trial sites will ensure not only the capacity to recruit study participants but also the capacity to manage (severe) adverse events and maintain compliance with good clinical and laboratory practice, including proper storage of acoziborole. Each one of the five clinical trial sites will have an on-site clinical trial investigator, who will be also in charge of enrolment in a number of other sentinel sites ( Figure 4).

Study samples will be shipped at least once a month to the reference laboratory in Kinshasa. No cold chain is planned at field level for the baseline sample. However, for any sample collected at month 4 post-acoziborole administration, a cold-chain at -20°C will be ensured at field level and throughout the storage and shipment process, for optimal RNA preservation, to verify the success of parasite clearance. At the INRB the collected specimens will be stored in the bio-bank, entered in the INRB data system and processed. The results will be made available to users via an automated online system (TrypElim, https://www.trypelim.org, based on libraries provided by Open Data Kit, ODK, https://getodk.org/ and the Iaso Platform, source code and open source licence available at Github: https://github.com/BLSQ/iaso/). 

### Laboratory procedures and interpretation of laboratory tests

To ensure proper monitoring of disease incidence and prevalence in the STROGHAT study, high sensitivity and specificity post-hoc molecular and immunological reference laboratory tests have been included in the diagnostic algorithm ^20– 22, 27– 30^. These tests will be performed at INRB in Kinshasa on samples collected in the field. The DBS will be used for immunological tests at the reference laboratory. Total nucleic acids will be extracted from the EDTA blood preserved in DNA/RNA shield buffer (Zymo Research, R1200-125) and tested in *Trypanozoon*-RT-qPCR multiplex ( Table 3).

**Table 3.** Specification of primers and probes used in *Trypanozoon*-RT-qPCR multiplex nucleic acid detection.

Name	Description	Oligonucleotide sequence (5’>3’)	Label	Reference
TBRF	Forward primer for TBR sequences	CGC AGT TAA CGC TAT TAT ACA	none	Van Reet *et al*., 2021
177 _T_-R	Reverse primer specific for 177-bp TBR group	GGA CCA TTA AAT AGC TTT GTT G	none	Van Reet *et al*., 2021
177 _T_-P	Probe specific for 177-bp TBR group	TGC CAT ATT AAT TAC AAG TGT GC	FAM, BHQplus-1	Van Reet *et al*., 2021
18S2-F	Forward primer for 18S rRNA	CCA ATC GGA CGC TCT CTT T	none	N’Djetchi MK, *et al*. 2024
18S2-R	Reverse primer for 18S rRNA	GTG GAG GCG TTG GTT CTA AT	none	N’Djetchi MK, *et al*. 2024
q18S2-P	Probe for 18S rRNA	TTG TGT TTA CGC ACT TGT CGT GGC	LGC Orange 560, BHQplus-1	N’Djetchi MK, *et al*. 2024
RP-F	Human RNAse P forward primer	AGA TTT GGA CCT GCG AGC G	none	Lu *et al*., 2020
RP-R	Human RNAse P reverse primer	GAG CGG CTG TCT CCA CAA GT	none	Lu *et al*., 2020
RP-P	Human RNAse P probe	TTC TGA CCT GAA GGC TCT GCG CG	Cy5, BHQplus-2	Lu *et al*., 2020

The role, oligonucleotide sequence, presence of a label and corresponding reference are listed for primers and probes used for nucleic acid detection by *Trypanozoon*-RT-qPCR multiplex in the STROGHAT study.

All individuals testing positive at baseline with either an immunological or molecular test, will be considered at risk of gHAT (parallel test result interpretation will optimize sensitivity). If included in the clinical trial and treated with acoziborole, a second blood specimen will be collected four months after acoziborole treatment in order to verify effective parasite absence four months post-treatment for individuals at potential risk. For determining gHAT prevalence throughout the study, maximum specificity will be preferred and test results will be interpreted serially, to avoid false positives, considering confirmed gHAT cases only those positive both in immunological and molecular tests at the reference laboratory, or testing positive on parasitology on the spot ( Table 2).

### The clinical trial implementation

**Figure 5.**
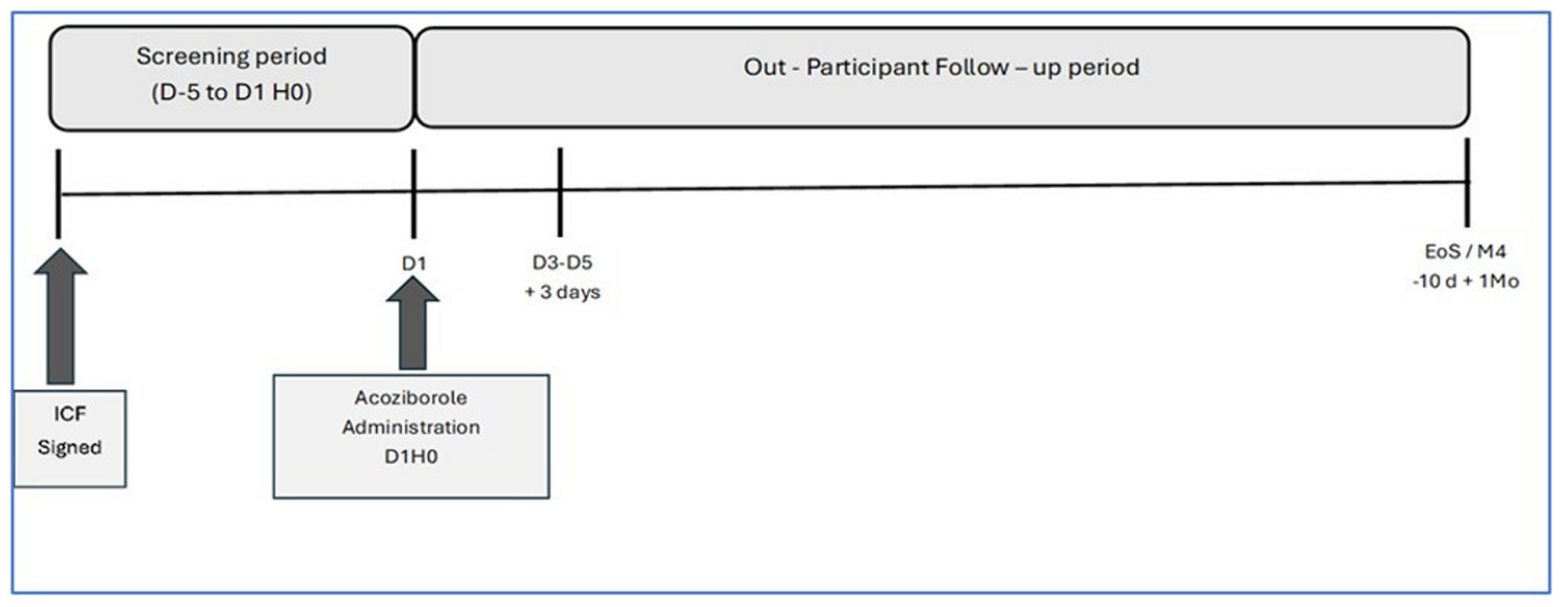
The STROGHAT clinical trial study design. The different key moments in the design of the STROGHAT clinical trial and the clinical trial timeline are shown. ICF: informed consent form; D: day; H0: administration time; EoS: end of study; M: month.

The duration of the clinical trial will be 3 years ( Figure 5, Table 4).

**Table 4.** The STROGHAT nested clinical trial Schedule of Events.

	Participants Follow-up			
	Screening D-5 to D1H0	Dosing D1H0	1st visit Post dosing D3- D5 (+3 days )	M4 visit (-10 days +1month (EoS)
Informed consent	X			
Pregnancy test	X			X
Demographics	X			
Medical History	X			
Concomitant medications	X		X	X
Clinical examination	X		X	X
Inclusion/Exclusion criteria	X			
Administration of acoziborole		X		
Collection of AEs (throughout part B)	X	X	X	X
Collection of blood samples for exploratory laboratory assessment				X

The Table summarizes the different actions to be taken and their timelines in the nested clinical trial. D: day; H0: administration time; M4: month 4; EoS: End of Study.

In principle all those testing positive on a screening test in the field (CATT or HAT Sero *K*-Set) are eligible for inclusion, i.e. treatment with acoziborole. Main exclusion criteria are age < 11 years, pregnancy or breast feeding, and being confirmed parasitologically on the spot, the latter category will receive standard of care treatment. Further details on inclusion and exclusion criteria are provided in Table 5.

**Table 5.** Inclusion and exclusion criteria for the STROGHAT clinical trial.

	Inclusion criteria	Exclusion criteria
1	Able to give signed informed consent (assent for adolescents)	Individuals with a positive parasitological exam on the spot at baseline
2	Both sexes	Participants previously treated for g-HAT or previously treated because of gHAT seropositive results
3	11 years of age or older and weight ≥30 kg	Pregnant women
4	CATT or RDT test positive	Breast-feeding women
5	Able to ingest oral tablets	Children ≥11 years but under 30kg body weight
6	Known address and/or contact details provided	Clinically significant medical condition
7	Able to comply with the schedule of follow-up visits and other study requirements	Jaundice
8	Agree not to take part in any other clinical trials during the participation in the clinical trial	Participants who are taking, or who are expected to need to start within 4 months, a medicine (including traditional or herbal) which may interact with acoziborole and which cannot be stopped or adjusted

Only individuals complying with all the inclusion criteria can participate in the STROGHAT nested clinical trial. Presence of one of the exclusion criteria excludes participation in the study.

Treatment will be provided by a study physician at the selected study sites. A study nurse will conduct a safety visit, at home or at the study site, 3–5 days and four months after treatment with acoziborole, to enquire about adverse events (AEs) ^31^. International standards will be ensured for AEs notification, follow-up, severity and causality assessment ^31, 32^. The village health workers or a designated person will be the liaison to ensure close follow-up of the clinical trial participants, encourage study adherence and inform the clinical trial investigators in case of safety concerns from the participants. 

### The clinical trial investigational product

Enrolled participants will receive acoziborole tablets of 320 mg: adults and adolescents ≥15 years 960 mg, children 11–14 years old (and ≥ 30 kg) 640 mg, in a single intake at study day 1 ^10^ (DNDi-OXA-05-HAT, ClinicalTrials.gov NCT05433350). Acoziborole will be managed as per good clinical practice (GCP) procedures and at controlled temperature not exceeding 30 °C.

Drug-drug interaction studies revealed interactions between acoziborole and several classes of drugs, acoziborole being an inhibitor of cytochrome CYP2D6 and an inducer of the cytochrome CYP3A4. Therefore, clinical trial participants will be asked not to take any medicine during the four months following acoziborole dosing without a medical consultation (including concomitant traditional medicine intake). Preclinical studies showed that acoziborole has no effect on fertility or foetal development. However, as a precaution in the absence of sufficient human pregnancy outcome data, women of childbearing potential will be required to use an effective form of contraception for the 4 months period after receiving acoziborole and pregnant and lactating women are excluded from the study.

### Data collection, data analysis and sample size

Data management processes will be set up in compliance with the International Council for Harmonization guideline for good clinical practice (ICH-GCP). We adopted the principles of data minimization, proportionality of data collection and pseudonymizing personal data. We adopted Privacy by Design in the data management setup and a Data Protection Impact Assessment will be performed prior to any personal data collection, in compliance with the EU GDPR regulation. Data security and confidentiality will be ensured.

The PNLTHA has been using an electronic health management information system, ‘Trypelim’ for data collection purposes since 2019. Individual data on active and passive screening and on treatment of gHAT are routinely entered. The STROGHAT epidemiological study will not introduce any additional data collection tool, but will rely on pseudonymized data extracts from Trypelim. Data will be extracted at 6, 12, 24, 36 and 48 months after the start of the study. The extracted dataset will contain variables such as age, sex, village name and location, date of screening, treatment taken and results of all screening and diagnostic tests performed, at field and referral laboratory level.

The clinical trial will use individual data collected through an electronic case report form, Medrio. Data collected will include participants demographic data, medical history, clinical examination and vital signs, child bearing potential, concomitant medications, acoziborole dose, adverse events, and child surveillance. Data will be accessible only to the clinical trial investigators and will be pseudonymized. An interim safety analysis (descriptive statistics) on participants treated with acoziborole is planned after approximately 1000 participants have been treated and been followed for four months.

The sample size for the clinical trial was determined by the epidemiological study sample size and on the assumed proportion of seropositive individuals that will be detected and treated. A total of around 2,500 seropositive individuals is expected. This, assuming a true incidence of severe related AE of 0.1%, would give a 92% chance of detecting a severe related AE ( Table 6) and will complement the available safety data ( *DNDI-OXA-04-HAT*, Clinicaltrials.gov NCT05256017 *)*.

**Table 6.** Chance of detecting one adverse event with various real incidences and sample sizes.

Real incidence (%)	Sample size	Chance of detecting at least one event
0.1% (1/1000)	900	59%
0.1%	1150	69%
0.1% *	1245	71%
0.1%	1500	77%
0.1%	1610	80%
0.1%	2000	86%
0.1%	2500	92%
0.1%	3000	95%
0,13% * (1,3/1000)	1245	80%
0.2% (2/1000)	900	83.5%
0.5% (5/1000)	900	98.9%
1% (10/1000)	900	99.99%

The Table illustrates what is the chance of detecting at least one adverse events with various disease incidences and sample sizes. * Corresponds to the current number of individuals exposed to acoziborole. The incidence is set at 0.13% to reach a 80% chance of detecting an event.

### Ethics and consent

The experimental protocol for this study has been designed in accordance with the general ethical principles outlined in the Declaration of Helsinki and ICH-GCP guidelines (ICH E6 R2) ^33^. The study has been approved by the following ethical committees: the Institutional Review Board of the Institute of Tropical Medicine in Antwerp (date: 15 January 2024, reference number: 1730/23), the Ethical Committee of the University of Antwerp Hospital in Antwerp (date: 04 March 2024, project ID: 6225) and the Comité National d’Ethique de la Santé, DRC (date: 17 February 2024, approval number: 512/CNES/BN/PMMF/2024).

The study will be conducted in compliance with general data protection regulation (GDPR), national regulations and GCP standards.

Taking into account the need for routine screening activities to continue without any impediments and given the expected individual and societal benefit of this study, a waiver for the absence of informed consent for the epidemiological study was requested and obtained from the above-mentioned ethical committees. A written individual informed consent (ICF) will be obtained from all participants in the nested clinical trial and will include blood sampling at the four-month visit which is not part of routine practice in the DRC. The ICF will be available in the local language and images will be available for clarification. An example of the ICF is available on open-access repository ^34^. The legal age of consent in DRC is 18. For study participants between 11 and 18 years old, informed consent of the parents or the guardian will be sought and participants will be asked for assent, unless they have more than 14 years old and are emancipated (Law 16/008 of 15 July 2016 amending and supplementing Law n. 87-010 of 1 August 1987 on the DRC Family Code in its articles 289-298).

## Discussion

The STROGHAT study will assess whether a strategy based upon extended treatment of parasitologically negative gHAT seropositive participants with acoziborole will succeed in eliminating *T.b.gambiense* from its human reservoir, and achieve the goal of interrupting transmission in a gHAT endemic focus, in line with the WHO elimination agenda ^7^. The study will also provide further safety data on acoziborole, assess the cost of the ‘screen and treat’ strategy and the performance of the screening and diagnostic tests in use. The STROGHAT study will be key to informing future elimination strategies and programs, by addressing many of the current challenges that could hinder the *T.b.gambiense* elimination goal, and by providing evidence about the effectiveness, safety and feasibility of the new strategy.

There are multiple reasons why the screen and treat approach tested in the STROGHAT study can be a gamechanger when it comes to interruption of transmission of *T.b. gambiense*. Firstly, we will expand treatment to all gHAT seropositive participants, overcoming the challenges linked to the suboptimal sensitivity of the current diagnostic algorithm ^8^. Additionally, the STROGHAT screen and treat strategy should improve acceptance and adherence to gHAT screening and treatment by reducing well known barriers such as fear of lumbar puncture and of the toxicity of treatment ^35, 36^.

Simplified and faster diagnostic processes, with treatment available on the spot and in a single dose, and very few side effects, could also encourage participation in screening activities by the high risk, hard-to-reach groups, that could be systematically missed by standard diagnostic algorithms ^35, 37^. STROGHAT will also address potential concerns of the affected populations about this new strategy. A qualitative study to assess acceptability was performed in the region prior to the start of the STROGHAT study, and regular qualitative assessments will be carried out during the study period. These will inform the community awareness and education interventions, and allow PNLTHA and its partners to consider adaptations of the screening program that could lever acceptance.

A potential concern of applying a strategy based on extended treatment of all individuals testing seropositive is the resulting massive overtreatment with a potential lower benefit/risk ratio in comparison with the current standard of care. There is, therefore, a need for very specific screening tests. WHO developed a target product profile (TPP) requiring a minimum specificity of 95% for a gHAT test to identify individuals to receive widened treatment ^38^. CATT appears to meet this target, for RDTs some uncertainty prevails ^22^. The STROGHAT study will provide additional safety data to support acoziborole use in a larger population at low risk of infection, at the same time it will provide additional data on the performance of the screening tests in use.

Additionally, despite not fully complying with WHO criteria and procedures for the verification of interruption of gHAT transmission ^6^, the STROGHAT study will evaluate absence of transmission on the basis of an expanded case definition. The case definition includes not only parasitologically confirmed cases but also parasitologically non-confirmed participants with both a positive molecular and immunological tests at referral laboratory level, thereby increasing its sensitivity.

There are a number of limitations to the STROGHAT study design. Because the gHAT prevalence level is already very low, the sample size that would be required to compare the intervention with standard of care was not deemed feasible. Therefore, a quasi-experimental design has been adopted, recognizing the limitations of such a design on the effectiveness assessment. We aim to demonstrate zero prevalence after three years of intervention, but this could be complicated by the fact that the current level of coverage of the population at risk by active and passive case detection is not entirely clear. There might be unrecorded gHAT cases, and, therefore, villages not appearing in the WHO lists 1 and 2, that could eventually be picked up in the survey in year 4.

There are also limitations to using parasitology as reference test to evaluate the specificity of the tests used in the STROGHAT study, although today there is no better alternative, and the same methodology has been applied elsewhere ^19, 27, 39^. In addition, because parasitology will be performed only on screening test positives, there is a risk of including false screening tests negatives in the denominator of specificity calculations and, at the same time, excluding them from examination and treatment. However, taking into account the very low gHAT prevalence expected, in combination with the high sensitivity of the screening tests reported elsewhere, those false negatives will be a negligible fraction of all the negative results observed ^25, 40^.

An additional limiting factor, not only to the STROGHAT study, but also to any possibility of reaching interruption of gHAT transmission as targeted by WHO, is the uncertainty concerning the role that infected animals could play in the gHAT transmission cycle ^2^. However, humans are assumed to be the major reservoir of gHAT and, were a supposed animal reservoir to have played a major role in gHAT transmission, we would in all probability not have reached the current low levels of transmission, since the interventions in place do not target any animal reservoir.

The STROGHAT study is also addressing key current and projected challenges. Assuming that ‘screen and treat’ will be the strategy applied in the future, a precise estimate of the true prevalence will be needed to allow for proper programmatic decision making. This could be a challenge, given the current low endemicity level, and the suboptimal performance of the screening and diagnostic tests in use. STROGHAT is addressing this by testing the feasibility of using high sensitivity and specificity immunological and molecular tests for post-hoc confirmation of all individuals seropositive at screening, to allow a very precise estimate of the true prevalence. This is in line with WHO recommendations, which have already set the expected performance of these tests ^41^. Additionally, there is still uncertainty about the performance of post-hoc tests ^22^, and STROGHAT will provide further evidence in this area.

If the screen and treat strategy tested in the STROGHAT study proves to be effective, safe and feasible, it may be assumed that, in future, there will be less need for complex parasitological confirmation techniques, which should result in a considerable simplification of current logistical procedures and a reduction in the need for experienced technicians at field level. The STROGHAT study will provide information on the costs of the so-called ‘screen and treat’ strategy, which could be of key importance to endemic countries and donors in evaluating the feasibility of implementing this new strategy.

The STROGHAT study is also addressing additional elements required to reach interruption of transmission, such as availability of funding, leadership, community understanding and political support. The STROGHAT study is designed and performed together with the PNLTHA and INRB, ensuring uptake at national level in the DRC, home to more than half of all gHAT cases reported in 2023 and previously ^42^. WHO and the main HAT donors have been involved in the study design. Results will be widely shared with affected communities, health authorities of DRC and other gHAT endemic countries, WHO, the donor community and the pharmaceutical industry. This will increase ownership of the elimination agenda by the affected communities and national health authorities, and possibly adoption of the tested strategy as part of the WHO and national policies to reach interruption of transmission at continental level. Evidence provided by the STROGHAT study will hopefully result in long-term commitments by donors for a disease that is currently no longer a major public health problem in affected countries, but has shown a tendency for resurgence multiple times in the past.

In conclusion, current developments in the gHAT field, including the steadily decreasing prevalence limited to a few hotspots within most endemic countries ^15^, suggest a promising possibility that interruption of transmission can be reached, if the proposed strategy proves effective, safe and feasible, and faces no major implementation hurdles. Nonetheless, even if interruption of transmission is achieved, post-elimination surveillance and vector control will be critical. Tsetse flies remain present in the environment, and the disease could potentially re-emerge from an animal reservoir, through human mobility of undetected cases, or from areas difficult to reach and to monitor ^43^.

## List of abbreviations

AE                     Adverse Event

CATT                Card Agglutination Test for Trypanosomiasis/T.b.gambiense

CI                      Confidence interval

CRF                   Case Report Form

DBS                   Dried blood spots

DDI                    Drug – Drug Interaction

DNDi                 Drugs for Neglected Diseases initiative

DRC                   Democratic Republic of Congo

EC                      Ethics Committee

EDCTP              European and Developing Countries Clinical Trial Partnership

GC(L)P              Good Clinical (Laboratory) Practice

GDPR                General data protection regulation

gHAT                 Gambiense human African trypanosomiasis

HAT                   human African trypanosomiasis

ICF                    Informed consent form

ICH-GCP          International Council for Harmonization guideline for good clinical practice

IDMC               Independent Data Monitoring Committees

INRB                Institut National de Recherche Biomédicale

IP                      Investigational Product

IRB                   Institutional Review Board

IRD                   Institut de Recherche et de Développement

ITM                  Institute of Tropical Medicine

mAECT            Mini anion exchange centrifugation technique

NECT               Nifurtimox Eflornithine Combination Therapy

PNLTHA          Programme Nationale de Lutte contre la Trypanosomiase Humaine Africaine

qPCR                Quantitative Polymerase Chain Reaction

RDT                  Rapid Diagnostic Test

SAE                  Serious Adverse Event

STROGHAT     Stop transmission of gambiense human African trypanosomiasis

TEAE               Treatment related emergent adverse events

TESAE             Serious treatment related emergent adverse events

TMG                Trial Management Group

TPP                  Target product profile

WHO               World Health Organization

## Declarations

### Ethics approval and consent to participate

The experimental protocol for this study has been designed in accordance with the general ethical principles outlined in the Declaration of Helsinki and ICH-GCP guidelines (ICH E6 R2) ^33^.

The study has been approved by the following ethical committees: the Institutional Review Board of the Institute of Tropical Medicine in Antwerp (date: 15 January 2024, reference number: 1730/23), the Ethical Committee of the University of Antwerp Hospital in Antwerp (date: 04 March 2024, project ID: 6225) and the Comité National d’Ethique de la Santé, DRC (date: 17 February 2024, approval number: 512/CNES/BN/PMMF/2024). The study will be conducted in compliance with general data protection regulation (GDPR), national regulations and GCP standards. Clinical trial registration: NCT06356974. Date of registration: April 4, 2024.

Taking into account the need for routine screening activities to continue without any impediments and given the expected individual and societal benefit of this study, a waiver for the absence of informed consent for the epidemiological study was requested and obtained from the above-mentioned ethical committees. A written individual informed consent (ICF) will be obtained from all participants in the nested clinical trial and will include blood sampling at the four-month visit which is not part of routine practice in the DRC. The ICF will be available in the local language and images will be available for clarification. An example of the ICF is available on open-access repository ^34^. The legal age of consent in DRC is 18. For study participants between 11 and 18 years old, informed consent of the parents or the guardian will be sought and participants will be asked for assent, unless they have more than 14 years old and are emancipated (Law 16/008 of 15 July 2016 amending and supplementing Law n. 87-010 of 1 August 1987 on the DRC Family Code in its articles 289-298). The study has been included in the Clinicaltrials.gov public registry (NCT06356974, April 4, 2024).

### Consent for publication

Not applicable. Manuscript describes a study protocol.

## Data Availability

No data are associated with this article. Zenodo: Example ICF STROGHAT. https://doi.org/10.5281/zenodo.14500360 ^34^. This project contains the following extended data: Example ICF STROGHAT.pdf Data are available under the terms of the Creative Commons Attribution 4.0 International licence. Zenodo: SPIRIT checklist for “The STROGHAT study protocol: an intervention study to evaluate safety, effectiveness and feasibility of treating gambiense HAT seropositive subjects with acoziborole.”. https://doi.org/10.5281/zenodo.14258618. Data are available under the terms of the Creative Commons Attribution 4.0 International licence.
