# Supplementary material for: The STROGHAT study protocol: An intervention study to evaluate safety, effectiveness and feasibility of treating gambiense HAT seropositive subjects with acoziborole
Source: Open Res Eur. 2025 Jan 24;5:23. [Version 1] doi: 10.12688/openreseurope.19077.1 (PMC11971624; doi:10.12688/openreseurope.19077.1)
Supplement: Peer review history

# Reviewer report: Reviewer response for version 1

**DOI:** 10.21956/openreseurope.20642.r52017

**Published:** 2025-04-04

**Authors:** Michael P. Barrett

**Affiliations:** 1 University of Glasgow, Glasgow, Scotland, UK

This article outlines the proposed study design of what could represent a transformative investigation into the prospect of changing treatment of human African trypanosomiasis, from one where it is necessary to perform a challenging diagnosis to one in which less potent testing can be followed by the safe provision of a new, orally available single dose drug that is curative of the disease whether in stage 1 or stage 2 (CNS involved).

 Generally the study is well designed and this manuscript well written. 

 I have two major points I'd wish to raise and then a number of smaller points and some typographical/grammatical suggestions.

 The major points are:

 1. With the growing evidence of trypanosomes being resident in skin, sometimes without accompanying sero-conversion, what plans are there to take this possibility into account should the evidence of this scenario reach the point where these cases risk diminishing the power of any prevalence work based on sero-conversion.

 2. Although the Discussion does point to key points for consideration in the deployment of the aspired Screen and Treat approach.  Are obvious pathways yet evident to how this will most likely be achieved?

 Minor points

 1.  Page 2.  Methods.  Line 8 estimate s not estimate

 2. Page 2. Plain Language Summary. Paragraph 1. line 8.  "has become" rather than "becomes"

 3. Paragraph 2.  Line 1 change "if" to "whether"

 4. Line 4.  add "in" after "help"

 5.  Line 6.  I'd suggest to add "where the disease has been prevalent in the past" after "Congo"

 Line 7.  After "drug" I'd add "that might have escaped detection in earlier clinical trials on smaller numbers of people"

 Page 4.  Introduction.  Paragraph 1.  Line 7. "non-specific" rather than "aspecific"

 Line 8.  "neurological" rather than "neurologic"

 Line 10.  Add "currently" in front of "diagnosed"

 Paragraph 3. Line 4.  Add a hyphen between nifurtimox eflornithine

 Line 7.  Change "would no longer be necessary" to "may no longer be necessary" (or add a qualifying phrase after "would no longer be necessary.....in such and such a circumstance..."

 Paragraph 4.  Line 2.  Change "highly effective" to "high efficacy"

 Line 9.  Change T.b. gambiense to T. b. gambiense (i.e. gap between T. and b.).  This needs correcting in multiple places throughout the text.

 Last line of that paragraph.  Change "of" the safety to "on the safety

 Study protocol

 Paragraph 2.  Line 7.  Usually it is "and/or" rather than "or/and"

 Page 5.  Table 1.  Column 4 in the DNDi-OXA-001 trial row,  part IV is referred to twice.  I think the second mention should be VI?

 Also Column 4 for the DNDi-OXA-02-HAT study, can the numbers of male and female be added?

 Page 6.  Figure 2 B.  Please increase the Font size for village names (preferably the in figure keys too)

 Page 7.  Line 16.  Add "million" after "2" (assuming it is 2 million and 2.5 million rather than "2" and 2.5 million!)

 Paragraph 4.  Line 7.  "stabilized WITH 1 mL.." rather than "on" 1 mL

  Paragraph 6 .  Are the enrollment years Jan 1 - Dec 31 for each of the study years?  Or were start and end dates different?  Its fine as written if that is the case, but if not the start and end dates should be given.

 Paragraph 7 (Outcome measures section)

 add "a measure of" after "study is.." giving "....study is a measure of the prevalence..."

 Paragraph 8 Line 2 year **s** 1,2,3

 Line 7.  sample **s**

 Line 14 "to conclude firmly" rather than to firmly conclude

 Paragraph 9. Please clarify a little more on why just one acoziborole tablet qualifies to be the study population when 2 or 3 tablets are the prescribed dosing.

 Page 9.  Line 3  "by the end of..."

 line 9  add "a" between "only" and "few"

 Associated with Paragraph 3.  Given the apparent batch variability within RDTs is any batch testing against set standards proposed to ascertain performance?

 Paragraph 4.  Line 5.  Is it -20oC or would other cold-chain methods (nitrogen or drug ice) be considered?

 Page 10.  Paragraph 1, last line tested "using" Trypanozoon rather than tested "in" Trypanozoon

 Paragraph 2.  line 8  Separate "For" from the preceding full stop.

 The last sentence is too long.  Can you change to

 For determining gHAT prevalence throughout the study, maximum specificity will be preferred and the test results will be interpreted serially to avoid false positive.  Only those positive both in immunological and molecular tests at the reference laboratory, or testing positive on the spot, will be considered as gHAT cases. 

 Last paragraph of the first column,  "In principle, all those...." (add a comma)

 Page 11.  Line 2  remove "s" from months (i.e. "...the 4 month period after..."

 Next section line 2.  "guideline **s** "

 Page 12.  Table 4.  I think worth adding in the legend to the Table that crosses indicate these items are included (because a cross often means "not" included)

 Table 5.  Item 5.  column 2 separate 30 from kg 

 Item 8.  Is there any way that possible interactions between acoziborole and herbal medicines can be known?

 Page 13.  Line 2.  Are there contingency plans if fewer than 2,500 seropositive individuals are found?

 Table 6.  Row 9.  Use a stop not a comma in 0.13%

 Table 6 legend.  line 2 "adverse event" not "adverse event **s** "

 Page 13. Column 2 line 5.  Add "an" before open-access

 Line 9 change "have" to "are"

 line 10  "Emancipated" seems a strange word in this context

 Discussion

 Paragraph 3. line 10 remove "the" before "community awareness"

 Page 14.  Paragraph 2.  line 7 remove "a" before "positive molecular.."

 Page 15.  Abbreviations.  Check T.b. gambiense here (and many places throughout)

 Hyphen between Niturtimox and Eflornithine

 TESAE  should this be Treatment related emergent serious adverse events (otherwise the abbreviated words are out of order)

 Page 17.  Can you confirm reference 34. makes a good connection on clicking the link? (otherwise difficult to trace)

Is the study design appropriate for the research question?

Yes

Is the rationale for, and objectives of, the study clearly described?

Yes

Are sufficient details of the methods provided to allow replication by others?

Yes

Are the datasets clearly presented in a useable and accessible format?

Not applicable

Reviewer Expertise:

Human African trypanosomiasis

I confirm that I have read this submission and believe that I have an appropriate level of expertise to confirm that it is of an acceptable scientific standard.

## References

[1] : The STROGHAT study protocol: An intervention study to evaluate safety, effectiveness and feasibility of treating gambiense HAT seropositive subjects with acoziborole. *Open Research Europe* .2025;5: 10.12688/openreseurope.19077.1 10.12688/openreseurope.19077.1 PMC1197162440191624

# Reviewer report: Reviewer response for version 1

**DOI:** 10.21956/openreseurope.20642.r50336

**Published:** 2025-03-10

**Authors:** Ifedayo Victor Ogungbe

**Affiliations:** 1 The University of Alabama in Huntsville College of Science (Ringgold ID: 307277), Huntsville, Alabama, USA

The STROGHAT study is an investigation aimed at addressing gambiense human African trypanosomiasis (gHAT).

 1. Its main objectives include achieving zero prevalence of *T.b. gambiense *infection within three years through a ‘screen and treat’ strategy using acoziborole, evaluating the cost-effectiveness of this approach, and assessing the performance of laboratory diagnostic tests. 

 2. To achieve these goals, the study will conduct a phase IIIb, one-arm, open-label trial to establish the safety of acoziborole in participants who have tested positive for gHAT but lack parasitological confirmation. The study will take place in the Democratic Republic of Congo (DRC) and will target regions that are at moderate to high risk for gHAT. Approximately 175,000 individuals are expected to be screened as part of this initiative.

 3. Overall, the study represents a good step toward eliminating gHAT by combining rigorous epidemiological analyses with safety assessments of acoziborole. Its targeted approach in high-risk areas aligns with the WHO's elimination roadmap and demonstrates a commitment to effective health strategies in endemic regions. This initiative has the potential to greatly improve health outcomes for vulnerable populations.

Is the study design appropriate for the research question?

Yes

Is the rationale for, and objectives of, the study clearly described?

Yes

Are sufficient details of the methods provided to allow replication by others?

Yes

Are the datasets clearly presented in a useable and accessible format?

Yes

Reviewer Expertise:

Drug Discovery

I confirm that I have read this submission and believe that I have an appropriate level of expertise to confirm that it is of an acceptable scientific standard.

## References

[1] : Efficacy and safety of acoziborole in patients with human African trypanosomiasis caused by Trypanosoma brucei gambiense: a multicentre, open-label, single-arm, phase 2/3 trial. *Lancet Infect Dis* .2023;23(4) : 10.1016/S1473-3099(22)00660-0 463-470 10.1016/S1473-3099(22)00660-0 36460027 PMC10033454
